# Sample Bias in Web-Based Patient-Generated Health Data of Dutch Patients With Gastrointestinal Stromal Tumor: Survey Study

**DOI:** 10.2196/36755

**Published:** 2022-12-15

**Authors:** Anne Dirkson, Dide den Hollander, Suzan Verberne, Ingrid Desar, Olga Husson, Winette T A van der Graaf, Astrid Oosten, Anna K L Reyners, Neeltje Steeghs, Wouter van Loon, Gerard van Oortmerssen, Hans Gelderblom, Wessel Kraaij

**Affiliations:** 1 Leiden Institute of Advanced Computer Science Leiden University Leiden Netherlands; 2 Department of Medical Oncology The Netherlands Cancer Institute Amsterdam Netherlands; 3 Department of Psychosocial Research and Epidemiology The Netherlands Cancer Institute Amsterdam Netherlands; 4 Department of Medical Oncology Radboud University Medical Center Nijmegen Netherlands; 5 Department of Surgical Oncology Erasmus Medical Center Rotterdam Netherlands; 6 Department of Medical Oncology Erasmus Medical Center Rotterdam Netherlands; 7 Department of Medical Oncology University Medical Center Groningen University of Groningen Groningen Netherlands; 8 Department of Methodology and Statistics Leiden University Leiden Netherlands; 9 Sarcoma Patient Advocacy Global Network Wölfersheim Germany; 10 Department of Medical Oncology Leiden University Medical Center Leiden Netherlands; 11 The Netherlands Organisation for Applied Scientific Research Den Haag Netherlands

**Keywords:** social media, patient forum, sample bias, representativeness, pharmacovigilance, rare cancer

## Abstract

**Background:**

Increasingly, social media is being recognized as a potential resource for patient-generated health data, for example, for pharmacovigilance. Although the representativeness of the web-based patient population is often noted as a concern, studies in this field are limited.

**Objective:**

This study aimed to investigate the sample bias of patient-centered social media in Dutch patients with gastrointestinal stromal tumor (GIST).

**Methods:**

A population-based survey was conducted in the Netherlands among 328 patients with GIST diagnosed 2-13 years ago to investigate their digital communication use with fellow patients. A logistic regression analysis was used to analyze clinical and demographic differences between forum users and nonusers.

**Results:**

Overall, 17.9% (59/328) of survey respondents reported having contact with fellow patients via social media. Moreover, 78% (46/59) of forum users made use of GIST patient forums. We found no statistically significant differences for age, sex, socioeconomic status, and time since diagnosis between forum users (n=46) and nonusers (n=273). Patient forum users did differ significantly in (self-reported) treatment phase from nonusers (*P=*.001). Of the 46 forum users, only 2 (4%) were cured and not being monitored; 3 (7%) were on adjuvant, curative treatment; 19 (41%) were being monitored after adjuvant treatment; and 22 (48%) were on palliative treatment. In contrast, of the 273 patients who did not use disease-specific forums to communicate with fellow patients, 56 (20.5%) were cured and not being monitored, 31 (11.3%) were on curative treatment, 139 (50.9%) were being monitored after treatment, and 42 (15.3%) were on palliative treatment. The odds of being on a patient forum were 2.8 times as high for a patient who is being monitored compared with a patient that is considered cured. The odds of being on a patient forum were 1.9 times as high for patients who were on curative (adjuvant) treatment and 10 times as high for patients who were in the palliative phase compared with patients who were considered cured. Forum users also reported a lower level of social functioning (84.8 out of 100) than nonusers (93.8 out of 100; *P=*.008).

**Conclusions:**

Forum users showed no particular bias on the most important demographic variables of age, sex, socioeconomic status, and time since diagnosis. This may reflect the narrowing digital divide. Overrepresentation and underrepresentation of patients with GIST in different treatment phases on social media should be taken into account when sourcing patient forums for patient-generated health data. A further investigation of the sample bias in other web-based patient populations is warranted.

## Introduction

### Background

Web-based patient forums provide patients with both emotional and informational support [1]. In recent years, social media (defined as a web-based communication channel where information and messages are exchanged) has also been investigated as a potential complementary information source for patient-generated health data, for example, for pharmacovigilance [2-6]. The main advantage of social media is that it offers uncensored information [7] in large quantities [8]. Moreover, patients are more likely to share information with fellow patients than with their physicians [9]. Thus, social media may contain information that is not collected in clinical trials or reported in spontaneous reporting systems.

Postmarket surveillance is necessary as clinical trials are of limited duration and suffer from sample bias; they often exclude older patients, patients with comorbidities, and pregnant women [10,11]. Current postmarket medication surveillance systems rely mostly on spontaneous reports of adverse events, medical literature, and observational databases. Most of these spontaneous reports are made by health professionals. In fact, in the Dutch surveillance system Lareb, only 26.3% of all reports between 2010 and 2015 were made by patients [12].

Reliance on spontaneous reports alone results in a severe underreporting of adverse drug responses (ADRs) [13]. According to the work by Lopez-Gonzalez et al [14], underreporting is associated with reporting of severe ADRs only, fear of ridicule for reporting suspected ADRs, lethargy, and indifference and complacency by professionals (ie, the idea that only safe drugs are allowed onto the market). Although previous work has shown that the ADRs reported on social media are often less serious than those reported via official channels, they do affect the quality of life of the patient [6]. In fact, social media would be able to provide a more patient-centric view of which ADRs are most salient to patients on a day-to-day basis [15].

However, researchers as well as patients have expressed concern about sample bias on social media [6,16-22]. Previous research on social media use in general shows that young people, women, and people of a higher socioeconomic class are generally highly represented [23-26]. Although there has been some work that shows that these differences persist over time [26,27], other work indicates that some factors such as age are becoming less influential as the overall adoption of social media is growing. According to a recent report of the Pew Research Centre, 72% of all Americans were using social media in 2021 including 45% of adults aged >65 years [28].

On the basis of studies of the general population of social media users [23-27], it appears that those demographic groups that consume more medication (ie, older patients, people of low socioeconomic status, and patients with chronic conditions) are generally not highly represented on social media platforms [14]. However, it remains unclear whether these findings generalize to the specific case of web-based patient-to-patient communication.

Although there is a large literature base on patient communication forums and the extraction of adverse drug effects, to date, the work on sample bias in web-based patient-to-patient communication is limited to 2 studies. Prior work on American patients with breast cancer [29,30] using action logs of forum activity in an artificial setting has shown that users are relatively more likely to be Caucasian than African American. No other significant demographic differences were found between users and nonusers. A more comprehensive overview of the literature on patient communication forums for patients with gastrointestinal stromal tumor (GIST) on broader topics than bias can be found in the recent work of den Hollander et al [31] and our own prior work [32].

Other studies addressed another bias that is relevant when mining social media for patient-generated health data: so-called activity bias [33] or the fact that only some users actively post messages. In this paper, we will use the term *passive users* for forum users that do not post messages and *active users* for forum users that do post messages. Passive users are also commonly referred to as *lurkers* in previous research. Among patients with breast cancer, Han et al [29] found that active users were more likely to be younger, Caucasian, living alone, and have a greater information need than passive users. Another study [34] specifically compared passive to active community members for breast cancer, arthritis, and fibromyalgia and corroborated that posters are younger on average. They also found that active users had a longer disease history and a higher self-reported mental well-being than passive users. In this paper, we do not compare active and passive users because of the small sample size.

As Baeza-Yates [33] noted, “any remedy of bias starts with awareness of its existence.” Thus, to provide a starting point for mitigating bias for the use of patient-generated health data from social media in the future, we conducted a survey to investigate sample bias in social media use among patients with GIST in the Netherlands relative to the survey sample. GIST is a rare form of cancer, which often has a long palliative care trajectory in which patients are treated with chronic, oral medication (tyrosine kinase inhibitors [TKIs]) for many years. If caught early, GIST can be cured. Treatment with TKIs can improve survival for patients with GIST both in adjuvant and palliative setting but often also lead to adverse drug events [31]. Patient reports from social media may be especially valuable for rare disorders where patients are sparse and spread out geographically.

### Objectives

In this study, we investigated (1) what proportion of patients have contact with fellow patients on social media, (2) why patients abstain from engaging with web-based patient communities, and (3) to what extent there are significant demographic and clinical differences between those that use social media to converse with patients and those that do not. This study did not assess general social media use but focused specifically on the web-based communication with other patients. We defined social media as a web-based communication channel where information and messages are exchanged. When referring to *web-based patient communities*, we mean web-based groups on social media where the main purpose of the group is for (certain) patients (eg, patients with breast cancer) to communicate with one another. We use the term web-based patient communities and patient forums interchangeably.

On the basis of general social media, we hypothesized that forum users will differ in demographic factors including age, sex, and socioeconomic status from nonusers. We also hypothesized that forum users will differ in marital status and have a lower level of social functioning than nonusers, in line with the social compensation model [35] (ie, those who have less real-life [offline] social support make more use of web-based digital communities). We also expect that forum users will differ from nonusers in their treatment status and that their symptom burden may be higher, whereas their global health scale may be lower. Overall, we expect patients with worse outcomes to be web-based more often to ask for and receive advice than their peers with better health outcomes.

## Methods

### Study Design and Participants

**Figure 1:**
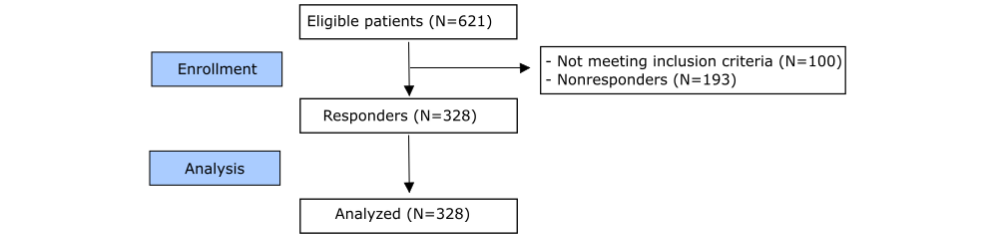
CONSORT (Consolidated Standards of Reporting Trials) flow diagram of response rate.

A cross-sectional study was conducted among Dutch patients with GIST aged ≥18 years at diagnosis, diagnosed between January 1, 2008, and December 31, 2018, in 5 GIST reference centers. Patients were selected from the Netherlands Cancer Registry (NCR), a population-based registry, which is maintained by the Netherlands Comprehensive Cancer Organization (in Dutch: Integraal Kankercentrum Nederland or IKNL) and collects patient and tumor characteristics on all newly diagnosed patients with cancer in the Netherlands. Exclusion criteria were cognitive impairment or being too ill at the time of the study according to advice from a (former) treating specialist. Eligible patients were invited by their (former) treating physician by a letter explaining the study. Upon consent of the patient, including permission to link the survey data with NCR data, patients could complete the survey on the web or on paper upon request. Refer to Figure 1 for a diagram of the response rate. Survey administration was done within the Patient-Reported Outcomes Following Initial Treatment and Long-term Evaluation of Survivorship registry [36], a data management system set up for the study of the physical and psychosocial impact of cancer and its treatment. Patient-Reported Outcomes Following Initial Treatment and Long-term Evaluation of Survivorship registry contains a large web-based component and is linked directly to clinical data from the NCR. Data were collected from September 2020 to June 2021.

### Ethics Approval

Ethics approval for the cross-sectional study was provided by the medical ethical committee of the Radboud University Medical Centre (2019-5888). According to the Dutch law, approval of one ethical committee for questionnaire research is valid for all participating centers. Patients gave informed consent, including permission to link the survey data with NCR data, before completing the survey.

### Survey

Participants completed questions regarding their participation in social media and web-based patient communities. These questions were developed by the authors. Respondents were asked whether and how patients use digital platforms to have contact with other patients. Possible answers (translated to English) were “Generic social media (like Facebook or Twitter),” “General forum or discussion group,” “Specific online patient forum,” “Other, namely...,” or “I do not use digital communication.” Patients were provided with the following definition for a digital medium (translated to English): a web-based communication channel where information and messages are exchanged between participants. Patients were allowed to give multiple answers.

Respondents having contact with other patients on the web were subsequently asked about their motivations for going on the web and about their frequency of posting messages. Both questions were adapted from a Dutch survey designed by van Uden-Kraan et al [34] in collaboration with medical experts and patient representatives. Survey respondents were allowed to provide multiple reasons for engaging with web-based forums as well as additional reasons in an open text field. Respondents who did not have contact with other patients on specific web-based patient forums were asked for their reasons for not doing so. Survey respondents were allowed to provide multiple reasons for abstaining from forum use as well as additional reasons in an open text field.

Demographic variables (ie, age, sex, and socioeconomic status) as well as clinical variables (ie, tumor type, tumor stage, time since diagnosis, whether surgery was performed, and whether targeted therapy was part of treatment) of survey respondents were collected from the NCR. Survey respondents were additionally asked about their marital status, their current treatment phase, whether they presently use medication, their most recent medication (if any), and the presence of the 14 comorbid conditions measured in the Charlson comorbidity index [37] (heart condition, stroke, high blood pressure, asthma, chronic bronchitis, chronic obstructive pulmonary disease, diabetes, stomach ulcer, liver disorder, blood disorder, thyroid disease, depression, arthritis, and back pain). Patients were allowed to fill in “Other” for the most recent targeted medication received for treating GIST. This option was intended for new or experimental TKIs, but because patients frequently used this option for other types of medication such as antacids, it was removed for post hoc analysis.

The options patients can choose for self-reported treatment phase are defined as follows: “Cured and not monitored” (“I am cured and no longer need to be monitored”) refers to patients who are considered cured after surgery with or without adjuvant imatinib, “On curative treatment” (“I am being treated and can still be cured”) refers to patients who are undergoing adjuvant imatinib treatment, “Follow-up after treatment” (“I am not being treated but am only being monitored”) refers to patients who are being monitored after surgery with or without adjuvant imatinib and are not undergoing treatment at this time, “On palliative treatment” (“I am being treated but cannot be cured”) refers to patients undergoing palliative treatment with thyroid kinase inhibitors, and “Best supportive care” (“I cannot be cured but am not being treated”) refers to patients who are palliative but are not receiving TKIs.

To measure overall health-related quality of life (QoL), social functioning, and symptom burden, participants completed the European Organisation for the Research and Treatment of Cancer Quality of Life Questionnaire C30 version 3.0 [38,39]. Health-related QoL was measured with 2 items on a scale from 1 to 7 (from “very poor” to “excellent”). Social functioning was measured with 2 items on a scale from 1 to 4 (1, “not at all”; 2, “a little”; 3, “quite a bit”; and 4, “very much”). Eight symptom-specific items were evaluated on the same scale (ie, dyspnea, pain, insomnia, appetite loss, nausea, constipation, diarrhea, and fatigue). Each symptom was measured with 1-3 items. The scores for a single symptom from multiple items were averaged. Symptom burden was measured by averaging the 8 symptom scales. For 17 respondents, symptom burden was not assessed, as there were missing data for at least one symptom. All scales were linearly transformed to a “0-100” scale in line with the standard scoring manual [40]. A higher score on the functional scales and global QoL means better functioning and QoL, whereas a higher score on the symptom scales means more complaints.

Any questions that were not previously validated were pretested with patients and changed according to their feedback (cognitive debriefing). The questionnaires cannot be shared because of copyright restrictions.

### Data Analysis

The reasons for abstaining and engaging with web-based patient-to-patient communication were analyzed manually by the first author. In total, 15.8% (52/328) of the cases contain missing data. As none of these cases are forum users, the data are not missing completely at random. As we do not observe any other patterns in the missing data that cannot be explained by the variables on which we have full information, the data are missing at random. As the missing data occur in multiple variables, we used Multivariate Imputation by Chained Equations [41,42] to impute these values, which is valid under the assumption of missing at random. We generated 20 imputed data sets that include all survey respondents (N=328).

We aimed to analyze whether there were statistically significant differences in demographic and clinical characteristics as well as the QoL measures between forum users and nonusers. For each imputed data set, a multiple logistic regression analysis was performed with forum use as the dependent variable and demographic and clinical factors as the independent variables (refer to Surveys). The effects of one variable on forum use are thus conditional on the other variables in the model. We report the average and SD of the 20 imputed data sets, as this provides a more reliable result than a single run. We use the mean as the average for all variables except the *P* value where we use the median [43].

For this analysis, the number of variables was restricted by the small size of the user population. We checked for multicollinearity using Variance Inflation Factor tests. If the Variance Inflation Factor value was >3, we removed one of the collinear explanatory variables. In total, we removed 2 variables accordingly: the most recent medication and whether the patient is on systemic treatment currently (“On systemic treatment currently”). Note that whether the patient received targeted therapy at some point in time (“Targeted therapy”) is included. Moreover, 2 categories of self-reported treatment phase, namely, on palliative treatment and on best supportive care, needed to be merged into one palliative category, as only one patient was receiving best supportive care.

Benjamini-Hochberg correction [44] was used to adjust for multiple testing (controlling the false discovery rate or type I errors at 0.05). Analyses were conducted using *statsmodels* (version 0.12.2) and *scipy* (version 1.4.1) in Python 3.7. Graphs were created with *plotly* (version 5.3.1) in Python 3.7.

## Results

### Participants

In total, 328 patients with GIST responded to the survey (response rate 64%). The median age of the participants was 67 (range 28-91) years, and 53.8% (174/328) of the participants were male (Table 1). On average, they had been diagnosed with GIST for 5 years, ranging from 1 to 12 years since diagnosis. In total, 49.3% (162/328) of the participants are in follow-up after treatment with curative intent, 18.5% (61/328) were considered cured and are not in follow-up, and 30.4% (100/328) receive systematic treatment either with curative (n=34) or palliative intent (n=66). Moreover, 1 patient received the best supportive care only.

Overall, 9 patients did not answer the question about forum use, and their forum use is thus unknown. Consequently, the sum of the reported numbers under forum use (Yes and No) does not equal the number reported for all respondents. The percentages were calculated based on the counts per category, that is, 54.9% (150/273) of nonusers are male.

**Table 1:** Demographic characteristics of survey respondents.

Demographic characteristic		All (N=328)	Forum user^a^	
			No (n=273)	Yes (n=46)
Age (years), median (range)		67 (28-91)	68 (28-91)	65 (47-83)
**Sex, n (%)**				
	Male	174 (53)	150 (54.9)	21 (45.7)
	Female	154 (47)	123 (45.0)	25 (54.3)
**Socioeconomic status, n (%)**				
	Low (1-3)	90 (27.4)	74 (27.1)	13 (28.3)
	Intermediate (4-7)	132 (40.2)	113 (41.4)	16 (34.8)
	High (8-10)	106 (32.3)	86 (31.5)	17 (37)
**Marital status, n (%)**				
	Married or living together	246 (7)	202 (74)	38 (82.6)
	Single	79 (24.1)	68 (24.9)	8 (17.4)
	Missing	4 (1.2)	3 (1.1)	0 (0)
Time since diagnosis (years), median (range)		5 (1-12)	5 (1-12)	5 (2-11)
**Tumor stage, n (%)**				
	I	121 (36.9)	109 (39.9)	8 (17.4)
	II	61 (18.6)	51 (18.7)	10 (21.7)
	III	66 (20.1)	53 (19.4)	10 (21.7)
	IV	55 (16.8)	38 (13.9)	16 (34.8)
	Missing	25 (7.6)	22 (8.1)	2 (4.3)
**Surgery, n (%)**				
	Yes	287 (87.5)	244 (89.4)	36 (78.3)
	No	41 (12.5)	29 (10.6)	10 (21.7)
**Targeted therapy, n (%)**				
	Yes	214 (65.2)	170 (62.3)	39 (84.8)
	No	114 (34.8)	103 (37.7)	7 (15.2)
**Self-reported current treatment status, n (%)**				
	Cured and not monitored	61 (18.6)	56 (20.5)	2 (4.3)
	On curative treatment	34 (10.4)	31 (11.4)	3 (6.5)
	Follow-up after treatment	162 (49.4)	139 (50.9)	19 (41.3)
	On palliative treatment	66 (20.1)	42 (15.4)	22 (47.8)
	Best supportive care	1 (0.3)	1 (0.4)	0 (0)
	Missing	4 (1.2)	4 (1.5)	0 (0)
**On systemic treatment currently, n (%)**				
	Yes	208 (63.4)^b^	181 (66.3)	25 (54.3)
	No	108 (32.9)	83 (30.4)	21 (45.7)
	Missing	12 (3.7)	9 (3.3)	0 (0)
**Most recent medication, n (%)**				
	Imatinib	178 (54.3)	140 (51.3)	31 (67.4)
	Sunitinib	9 (2.7)	7 (2.6)	2 (4.3)
	Regorafenib	6 (1.8)	4 (1.5)	2 (4.3)
	Other	15 (4.6)	8 (2.9)	4 (8.7)
	No therapy	114 (34.8)	103 (37.7)	7 (15.2)
	Missing	14 (4.3)	11 (4)	0 (0)
**Number of comorbid conditions, n (%)**				
	0	109 (33.2)	92 (33.7)	14 (30.4)
	1	71 (21.6)	59 (21.6)	10 (21.7)
	2+	146 (44.5)	120 (44.0)	22 (47.8)
	Missing	2 (0.6)	2 (0.7)	0 (0)
Global health scale (0-100), mean (SD)		78.6 (18.1)	79.0 (17.7)	76.1 (20.1)
Symptom burden (0-100), mean (SD)		12.1 (12.8)	11.4 (12.6)	15.6 (13)
Social functioning (0-100), mean (SD)		92.4 (18.9)	93.8 (17.1)	84.8 (26)

^a^Nine participants did not answer this question.

^b^It appears that patients who are currently being monitored may have misunderstood this question, inflating the number of patients who are currently on targeted medication for gastrointestinal stromal tumor.

### Social Media Use

Among the participating Dutch patients with GIST, 81% do not have contact with other patients via any social media platform (Table 2). We distinguished between specific social media, such as patient forums, and general social media, such as Twitter or Facebook. Although it is possible for patient communities to exist as groups on general social media platforms (in fact: the biggest GIST forum is a Facebook group), general social media refers to communication with peers outside of GIST-specific communities on these general social media platforms. Of the patients who communicate with peers via social media, the majority (46/59, 78%) make use of specific web-based patient forums focused on GIST. Only 6 respondents make use of general social media platforms to communicate with other patients with GIST, and only 7 respondents use more general cancer-related forums or discussion groups for this purpose.

**Table 2:** Descriptive statistics for the use of social media to have contact with other patients (N=328).

Which of the following digital media do you use to have contact with other patients?^a^ (Indicate all that apply)	Values, n (%)
General social media (such as Facebook or Twitter)	6 (1.8)
General cancer-related forum or discussion group	7 (2.1)
GIST^b^-specific web-based patient forum	46 (14)
Any social medium	59 (18)
None or via another medium than social media	265 (80.8)
Missing	4 (1.2)

^a^Respondents can give multiple answers to this question.

^b^GIST: gastrointestinal stromal tumor.

### Reasons for Abstaining From Web-Based Patient-to-Patient Communication

Table 3 presents the reasons the 265 nonusers report for not using any digital medium to communicate with fellow patients. Patients were allowed to report multiple reasons. A total of 20 patients did not fill in the question. The most common reason reported for abstaining from using a digital medium to communicate with peers was that they felt no need to do so (78/265, 29.4%), followed by finding it too confronting (33/265, 12.5%) and not knowing where to find web-based communities (30/265, 11.3%). Only 8 participants reported not using social media to communicate with other patients because they lacked the skills or access to do so.

**Table 3:** The reasons nonusers report for not using social media to communicate with other patients (N=265).

Self-reported reason^a^	Values, n (%)
Feel no need to communicate (digitally) with other patients	78 (29.4)
I find it too confronting or burdensome	33 (12.5)
I do not know where to find online communities	30 (11.3)
There are too many negative comments	26 (9.8)
I do not have the time	23 (8.7)
The information shared is useless or less valuable	20 (7.5)
I communicate with enough patients personally or via another nondigital medium	18 (6.8)
I do not use social media, lack a computer or digital skills, or do not like obtaining information digitally	8 (3)
I obtain sufficient information via my medical specialist or by searching online	7 (2.6)
I no longer have symptoms or do not like to consider myself a patient	5 (1.9)
I have privacy concerns	3 (1.1)
They do not exist in my language	2 (0.8)
No particular reason	1 (0.4)
Missing	20 (7.5)

**^a^**Multiple answers were possible.

### Reasons for Engaging With Patient Forums

Survey respondents most frequently used patient forums to communicate with other patients. The number of survey responders that made use of other web-based platforms was too small to analyze how they compare with nonusers. Thus, we will focus on analyzing the sample bias of GIST-specific patient forums. Hereafter, when we refer to “forum users,” we mean users of GIST-specific patient forums.

Table 4 presents the reasons users reported for engaging with a disease-specific patient forum. The most prevalent reasons were having a question about their illness (18/45, 40%), having heard new information about their illness (18/45, 40%), and being curious about how the other members are doing (16/45, 36%). Another prevalent trigger was experiencing new symptoms (14/45, 31%).

**Table 4:** The reasons users report for visiting the patient forum (N=45).

Self-reported reason^a^	Value, n (%)
When I have a question about my illness	18 (40)
When I have heard new information about my illness	18 (40)
When I am curious about how other members are doing	16 (36)
When I observe new symptoms	14 (31)
When I have a lot of symptoms	6 (13)
When I feel insecure	5 (11)
Before making a medical choice	4 (9)
Because I enjoy the company	4 (9)
Because other members expect me to be there	2 (4)
When I feel lonely	1 (2)
It is part of my daily routine	1 (2)
I never use the forum anymore	1 (2)

**^a^**Multiple answers were possible.

### Characteristics of the Patient Forum Users

In total, 85.8% (273/328) of the participants were not making use of specialized GIST patient forums (Table 1). The difference in model fit between the multiple logistic regression model and the null model was found to be statistically significant in all 20 imputed data sets (likelihood ratio [LR]=47.0, SD 1.48, *df*=20; *P*<.001). LR tests between the full model and the full model without the variable were used to test significance of individual variables.

Table 5 reports the average results of 20 runs of multiple logistic regression models of which factors influence forum use. Our analysis shows that self-reported treatment status differs significantly between forum users and nonusers for each run (LR=10.6; *P=*.001). The odds of being on a patient forum were 2.8 times as high for a patient who is being monitored compared with a patient who is considered cured. The odds of being on a patient forum were 1.9 times as high for patients who were on curative (adjuvant) treatment and 10 times as high for patients who were in the palliative phase compared with patients who were considered cured.

We did not find significant differences between forum users and nonusers for other disease-related characteristics when they were adjusted for covariates. We also did not find significant differences in key demographic variables such as age, sex, socioeconomic status, and marital status. However, we did find a significant difference in level of social functioning in 7 of 20 runs (LR=6.8; *P*=.008). Forum users on average reported a lower level of social functioning than nonusers (84.8 vs 93.8 out of 100). These scores were normalized according to the scoring manual [40]. Converting the normalized values back to the mean raw score gives a 1.19 for forum users and a 1.46 for nonusers, where 1 translates to the highest possible value for self-reported social functioning on the survey items.

**Table 5:** Average results (with SD) of a logistic regression of demographic and clinical characteristics of patient forum users and nonusers using Multivariate Imputation by Chained Equations with 20 runs.

			Coefficient, mean (SD)		SE, mean (SD)		*df* ^a^		LR, mean (SD)		*P* value, median (SD)		Odds ratio				
													5% (SD)		Mean (SD)		95% (SD)
Intercept			−2.80 (0.54)		2.08 (0.03)		N/A^b^		N/A		N/A		N/A		N/A		N/A
Age			−0.02 (0.004)		0.02 (0.0002)		1		1.32 (0.54)		.26 (.10)		0.95 (0.004)		0.98 (0.004)		1.02 (0.004)
Sex			0.62 (0.04)		0.37 (0.004)		1		2.86 (0.35)		.09 (.02)		0.90 (0.03)		1.86 (0.07)		3.86 (0.16)
**Socioeconomic status**			N/A		N/A		2		1.37 (0.49)		.25 (.08)		N/A		N/A		N/A
	Low (1-3)^c^	N/A		N/A		N/A		N/A		N/A		N/A		N/A		N/A	
	Intermediate (4-7)	−0.39 (0.10)		0.44 (0.006)		N/A		N/A		N/A		0.20 (0.03)		0.68 (0.07)		1.62 (0.16)	
	High (8-10)	0.05 (0.10)		0.44 (0.005)		N/A		N/A		N/A		0.45 (0.04)		1.06 (0.10)		2.50 (0.26)	
Marital status			−0.32 (0.09)		0.47 (0.006)		1		0.52 (0.25)		.47 (.11)		0.29 (0.04)		0.73 (0.06)		1.82 (0.06)
Time since diagnosis			0.02 (0.02)		0.07 (0.001)		1		0.12 (0.16)		.85 (.15)		0.88 (0.02)		1.02 (0.02)		1.17 (0.02)
Tumor type			0.57 (0.06)		0.38 (0.003)		1		2.29 (0.52)		.13 (.04)		0.84 (0.05)		1.77 (0.11)		3.70 (0.24)
**Tumor stage**			N/A		N/A		3		2.60 (0.92)		.12 (.07)		N/A		N/A		N/A
	I^c^	N/A		N/A		N/A		N/A		N/A		N/A		N/A		N/A	
	II	0.51 (0.13)		0.55 (0.009)		N/A		N/A		N/A		0.57 (0.07)		1.67 (0.21)		4.89 (0.63)	
	III	0.21 (0.21)		0.63 (0.01)		N/A		N/A		N/A		0.37 (0.09)		1.27 (0.29)		4.31 (0.94)	
	IV	0.86 (0.17)		0.66 (0.01)		N/A		N/A		N/A		0.66 (0.12)		2.41 (0.43)		8.83 (1.61)	
Surgery			0.04 (0.12)		0.57 (0.01)		1		0.05 (0.10)		.89 (.10)		0.34 (0.04)		1.05 (0.12)		3.24 (0.42)
Targeted therapy			0.12 (0.10)		0.57 (0.01)		1		0.07 (0.08)		.83 (.10)		0.37 (0.03)		1.13 (0.11)		3.49 (0.38)
**Self-reported current treatment status**			N/A		N/A		3		10.67 (1.10)		.001^*^ (<.001)		N/A		N/A		N/A
	Cured and not monitored^c^	N/A		N/A		N/A		N/A		N/A		N/A		N/A		N/A	
	On curative treatment	0.59 (0.26)		1.07 (0.05)		N/A		N/A		N/A		0.23 (0.04)		1.86 (0.45)		15.56 (4.65)	
	Follow-up after treatment	1.03 (0.26)		0.87 (0.06)		N/A		N/A		N/A		0.52 (0.08)		2.88 (0.69)		16.18 (5.10)	
	Palliative	2.29 (0.23)		0.97 (0.06)		N/A		N/A		N/A		1.50 (0.23)		10.11 (2.21)		68.68 (19.84)	
**Number of comorbid conditions**			N/A		N/A		2		0.42 (0.26)		.53 (.14)		N/A		N/A		N/A
	0^c^	N/A		N/A		N/A		N/A		N/A		N/A		N/A		N/A	
	1	0.28 (0.11)		0.50 (0.007)		N/A		N/A		N/A		0.50 (0.06)		1.33 (0.14)		3.51 (0.36)	
	2+	0.21 (0.08)		0.45 (0.005)		N/A		N/A		N/A		0.51 (0.04)		1.23 (0.09)		2.99 (0.24)	
Global health scale or QoL^d^			0.03 (0.002)		0.01 (0.0001)		1		4.38 (0.69)		.04 (.02)		1.00 (0.002)		1.04 (0.002)		1.06 (0.002)
Symptom burden			−0.0003 (0.005)		0.02 (0.0004)		1		0.09 (0.10)		.83 (.11)		0.96 (0.006)		1.00 (0.005)		1.04 (0.005)
Social functioning			−0.03 (0.002)		0.01 (0.0002)		1		6.87 (0.90)		*.008* (.005)		0.96 (0.002)		0.98 (0.001)		0.99 (0.002)

^a^There are separate *df* values for each variable depending on the number of categories in that variable.

^b^N/A: not applicable.

^c^These categories were taken as the reference categories for calculating the influence of different categories of the variable on forum use

^d^QoL: quality of life.

*Italicized values indicate statistically significant values.

## Discussion

### Principal Findings

A survey was conducted among 328 patients with GIST in the Netherlands. Our results show that most survey respondents do not have contact with other patients via social media. They indicate a large heterogeneity of reasons of why they abstain from doing so, with the most prevalent being they feel no need, find it too confronting, or do not know where to find such web-based communities. Of the minority who do use social media for this purpose, most use disease-specific patient forums. The most prevalent reasons for accessing a patient forum are (1) having a question about their illness, (2) having heard new information, (3) experiencing new symptoms, or (4) wondering how other patients are doing. Patient forum users differ significantly in (self-reported) treatment phase from nonusers. Patients in the palliative phase are 10 times more likely to be forum users than patients who are cured. Patients who are monitored approximately 3 times and patients undergoing curative treatment approximately 2 times are more likely to be users than cured patients. For 7 of 20 data imputations, forum users also have a significantly lower level of social functioning.

### Comparison With Existing Literature

In contrast to the general population of social media users, patient forum users do not appear to differ in age, sex, and socioeconomic status from nonusers. On the one hand, this may be an effect of the increasingly more widespread adoption of social media. This idea is supported by the small number of patients that indicate they lack the skills or access to be on social media (8/265, 3.3%). On the other hand, it is also possible that there is less demographic bias on patient forums than in general social media. This may be related to the widely different goals that users have with their participation. Although a feeling of community and social support may overlap, patients report motivations such as questions around their illness and the experience of new symptoms that normal social media users are unlikely to share.

Prior work [29] on forum use among patients with breast cancer did not find significant differences between forum users and nonusers in terms of clinical characteristics, that is, stage of cancer and QoL. We similarly did not find any significant differences for these characteristics, although we did find significant differences for clinical characteristics that prior work did not investigate, that is, treatment phase. Prior work also found that among patients with breast cancer, nonusers and passive users had greater offline social support than posters. Their results supported the social compensation model [35], that is, those who have less real-life (offline) social support use and engage on the web with digital communities. The lower offline support of forum users compared with nonusers in our data also supports this theory. However, passive users appear to have a lower offline support than active users among patients with GIST. This would support the competing theory: the social engagement model [45], that is, those that have more social resources will use and benefit from web-based social communities more. Consequently, our data offer support for the social compensation model for those who use a forum (ie, those with less real-life support are more likely to be on a forum) and social engagement theory for those who actually actively engage with the forum community (ie, users with sufficient social resources will be active and benefit more). Demographic differences in terms of age, marital status (ie, living alone or not), and disease duration between passive and active users that were found in previous work were not evident from our data.

### Limitations

First and foremost, we only studied a specific patient population in a single country, and thus, further research is needed to elucidate to what extent our results are generalizable. Patients in other countries may have lower digital access or skills or may not wish to use social media for patient-to-patient communication for other reasons (eg, other privacy laws or country-specific customs).

Our choice of patients with GIST as a target population may also impact to which disorders our results generalize to. Patients with GIST have a median age of mid-60 years [46], meaning that it is on average an older population than the general population that is often studied for social media use. Our results may consequently also generalize better to conditions that are prevalent in an older population. GIST is also characterized by a long palliative phase in which patients receive treatment. Thus, our results may also generalize better to conditions that similarly have a long treatment duration (eg, metastasized breast cancer). As GIST is a rare type of cancer, our results may also generalize better to rare conditions than common conditions. Further research into other patient populations should be able to provide more insight into the differences in forum use between rare conditions and common conditions. The fact that GIST is a rare condition makes it an interesting first case. Patient-generated health data from social media are particularly promising for rare conditions because of their dispersed patient communities and the scarcity of research [47].

A second limitation of this study is the small sample size. Among the 328 respondents, only 46 (14.1%) indicate that they make use of patient forums. Nonetheless, given the low incidence of GIST at 12.7 per million [48], this is a substantial number of participants. A third limitation is the sample bias of the survey itself. There may be 2 underlying factors, namely, selection bias and responder bias. Patients who were too ill or had cognitive impairment were excluded, leading to selection bias. A nonresponder analysis was conducted using the database of the NCR to assess the extent of the responder bias. After correcting for multiple testing, no significant differences were found in terms of age, sex, socioeconomic status, time since diagnosis, tumor stage, and primary treatment between responders and nonresponders. Moreover, it was possible to fill in the survey on paper, which prevents the exclusion of less digitally adept patients on these grounds.

### Future Work and Recommendations

On the basis of this work, a number of recommendations can be made. First, of the possible digital resources that can be used to source complementary real-world evidence, for instance, for pharmacovigilance, patient forums should be preferred over other social media. Our results reveal that patients with GIST strongly prefer disease-specific patient forums over general social media for communicating with fellow patients. However, most research in this field currently focuses on general social media such as Twitter [4,5]. Our results are in line with previous work that estimates ADR reports to be more prevalent in patient forums than on Twitter [49].

Although we find that there is sample bias in patient forum users and, thus, the sample is not wholly representative for the patient population, sample bias is also a concern for other sources of patient reports. Understanding which patients are overrepresented and underrepresented on web-based forums is the first step to using web-based patient reports as a complementary resource, for instance, for pharmacovigilance, which is seen as a realistic first use case. For pharmacovigilance specifically, it is not of great concern that patients who are considered cured and not undergoing treatment currently are underrepresented. Future work into comparing the sample bias of clinical trials with that of web-based patient forums would be beneficial to further explore its complementary value in detail. It would also be valuable to gain more insight into the different types of forum users.

Second, it may be beneficial to create awareness among medical professionals that patients are more likely to search for information in web-based patient communities when they have questions, have been given new information, or have new symptoms. Medical professionals could try to aid patients in their information need by pointing them toward such resources in these cases. This may also take away the barrier mentioned by patients that they do not know where to find such web-based communities.

Third, future work into the sample bias of patient forums for other patient populations is necessary, as this study was limited to a single population in a single country. Nonetheless, our work is a stepping stone toward dissuading the concerns that researchers have expressed regarding the sample bias of social media [6,16-22] by unraveling on which characteristics users differ significantly from the overall patient population. Future work could also investigate how compensatory measures can be implemented to statistically correct for sample bias. As these factors may not be known for the participants of a forum, it would also be worthwhile to consider to what extent correcting for sample bias is possible without this information.

### Conclusions

In this study, we investigated how representative participants in patient forums are for the general patient population by conducting a survey among patients with GIST in the Netherlands. We found statistically significant differences in terms of treatment phase and offline social support between forum users and nonusers. The consequent overrepresentation and underrepresentation of certain types of patients should be considered when sourcing patient forums for patient-generated health data. As our study was limited to a single patient population, a further investigation of the sample and activity bias in other web-based patient populations is warranted. Sample bias is inherent to any information source, and only through awareness of these biases can these resources be used as a source for complementary real-world evidence in the future.

## Abbreviations

ADR: adverse drug response
GIST: gastrointestinal stromal tumor
LR: likelihood ratio
NCR: Netherlands Cancer Registry
QoL: quality of life
TKI: tyrosine kinase inhibitor

## Data Availability

The data are not publicly available.
